# Intended and Unintended Impacts of ‘4+7’ Volume-Based Drug Procurement on the Use of Drugs in China: A Natural Experimental Study

**DOI:** 10.3390/healthcare13060686

**Published:** 2025-03-20

**Authors:** Dantong Zhao, Zhongliang Zhou

**Affiliations:** 1 School of Humanities and Social Science, Chang’an University, Xi’an 710064, China; zhaodt1224@163.com; 2 School of Public Policy and Administration, Xi’an Jiaotong University, No. 28 Xianning West Road, Xi’an 710049, China

**Keywords:** volume-based drug procurement, ‘4+7’, volume, expenditure, China

## Abstract

**Background:** Volume-based drug procurement is regarded as a pharmaceutical cost-containment measure in healthcare provision globally. The Centralized Volume-Based Drug Procurement (CVBDP) launched in March 2019 in China, also known as the ‘4+7’ policy. 11 cities, including Xi’an city in Shaanxi Province, were set up as pilots. This study aims to examine the intended and unintended impacts of the ‘4+7’ policy on the use of original and generic drugs in city-level and county-level hospitals in Shaanxi, China. **Methods:** The data used in this study came from the Shaanxi Drug and Apparatus Centralized Procurement Platform (SDACPP). In total, 111,999 drug procurement order records of 118 policy-related drugs (including 25 ‘4+7’ policy-list drugs and 93 alternative drugs by generic name) from April 2017 to November 2019 were included in analyses. Policy-list drugs were divided into bid-winning and non-winning drug products. The volume and the expenditure of the drugs served as the outcome variables, measured by Defined Daily Doses (DDDs) and Chinese yuan (CNY), respectively. A difference-in-differences (DID) approach was used to estimate the policy’s net effect. **Results:** After the ‘4+7’ policy, the volume of bid-winning, policy-list, and policy-related drugs increased. An unexpected increase in volume was observed among alternative drugs, especially original drugs in city-level hospitals. The expenditure of policy-list and non-winning drugs declined, whereas that of alternative drugs unanticipatedly increased. Changes in volume and expenditure were both greater in generic drugs and in city-level hospitals, compared to their original and county-level hospital counterparts. **Conclusions:** Our findings highlight the positive effects of the ‘4+7’ policy on generic drug substitution and pharmaceutical expenditure containment, which are greater in city-level hospitals. The unanticipatedly incremental volume of original drugs in city-level hospitals suggests the potential risk of the poor quality of bid-winning drugs, lower compliance with bid-winning drugs among patients, or physicians’ profit-seeking behaviors in urban areas. More regulations and supervisions for the prescription and financial incentives of physicians are needed to address these concerns.

## 1. Introduction

The global demand and utilization of health services has increased dramatically in recent years, especially pharmaceuticals, which are regarded as the most important health component [1]. In China, the costs of pharmaceuticals accounted for 41.56% of the total health expenditures in 2008 [2], and they decreased to 32.73% in 2018 [3]. However, this figure is still much higher than that of other Asian countries, such as Japan (17.80%) and Korea (20.20%), as well as the Organization for Economic Cooperation and Development (OECD) countries’ average (16.29%) [4]. Many countries are looking for effective containment strategies for escalating pharmaceutical costs. Various policies focusing on the pharmaceutical supply chain were implemented, including the pharmaceutical industry, wholesalers and retailers, and prescribers and consumers [5]. Since the early 1990s, the Chinese government has implemented a price cap policy by directly setting the price ceiling for each individual brand of drug. But such efforts were not as effective as anticipated, and it was abolished in 2015, which might be attributed to the irrational cost-plus pricing method, the lack of transparency regarding the inputs and costs for price determination, and the profit-seeking behaviors of physicians on 15% markups from drug sales. In 2009, the zero-markup policy was put forward, where all public hospitals were prohibited from adding additional retail markups from the procuring price. Although this effort has been proven through a reduction in drug prices and drug expenditure [6], it created new problems, for instance, high wholesale prices, monopoly pricing, and shortages [7]. Concurrently, centralized drug procurement at a provincial level was carried out. It became the dominant pattern of drug procurement in hospitals in China. However, the issue of inflated drug prices, drug rebates, drug shortages, etc., has been widespread [8]. It is mainly attributed to the absence of a link between the prices and volumes of the purchased drugs [9].

### 1.1. Overview of ‘4+7’ Volume-Based Drug Procurement in China

Volume-based drug procurement is a global common practice to lower pharmaceutical prices by creating economies of scale and improving purchasing power [10]. After years of reform attempts, a novel centralized drug procurement named ‘Centralized Volume-Based Drug Procurement (CVBDP)’ was announced by the General Office of the State Council of the People’s Republic of China in January 2019 [11]. It aims to lower drug prices, alleviate the drug burden, regulate the order of drug circulation, and standardize prescriptions. In this pilot program, 11 cities including 4 municipalities (i.e., Beijing, Tianjin, Shanghai, and Chongqing) and 7 sub-provincial cities (i.e., Shenyang, Dalian, Xiamen, Guangzhou, Shenzhen, Chengdu, and Xi’an) in China were selected as pilot cities, also known as the ‘4+7’ policy. Unlike previous centralized drug procurement at a provincial level, the ‘4+7’ policy, which is organized by the central government and based on price–volume agreement (PVA), lies at the core of ‘volume for price’ and ‘recruitment and procurement integration’. The Joint Procurement Office led by the National Healthcare Security Administration (NHSA) determines the drug variety first, named ‘4+7’ policy-list drugs. Only original drugs and generic drugs that have previously passed the Generic Consistency Evaluation (GCE) are eligible to participate in procurement. Then, each public healthcare institution in the 11 pilot cities is required to submit the annual agreed procurement volume of the ‘4+7’ policy-list drugs to the NHSA. The agreed procurement volume is the predicted annual purchase volume of a certain drug (by generic name) estimated according to the use volume in the last year. The ‘4+7’ policy integrates the pharmaceutical procurement volume of public healthcare institutions in 11 pilot cities, which accounts for approximately 60–70% of the total annual drug use of these institutions. The bidding enterprise makes a quotation according to the specific quantity of each drug. Then, the Joint Procurement Office determines the purchase price of each size of drug through bidding or negotiation. Under the theory of economies of scale, the drug manufacturers reduce the drug price and obtain a larger market [12]. From the perspective of economics, CVBDP of the nature of ‘bulk purchasing’ forms about 70% of the ‘Monopsony’ of the NHSA, enhancing the negotiation power of healthcare institutions and payers. Through the linkage of price, volume, and proportion, the pharmaceutical price reduction and pharmaceutical costs are controlled, and the medical insurance fund savings are likely to be achieved. Eventually, 25 bid-winning drugs were successfully announced in the ‘4+7’ list, with an average price reduction of 52% [13].

### 1.2. Original and Generic Drugs

The development of new drugs requires massive amounts of time, money, and efforts [14]. The process is complex and costly. It generally takes 10 to 15 years, and its costs range from USD 800 million to USD 2 billion to obtain a new drug to market [15]. Generics are defined as drug products that are identical or bioequivalent to brand/reference listed drug products in every aspect including the dosage form, safety, strength, route of administration, quality, performance characteristics, and intended use [16]. Taking the lack of necessity to recoup research and development costs, payer pressures, and market competition into consideration, generic drugs can be priced as low as 2% to 10% of the pre-patent loss price and are typically 20% to 90% cheaper than their original counterparts [17]. Thus, the use of generic drugs is more prevalent in recent years, primarily as a pharmaceutical cost-containment measure in healthcare provision [15]. It is worth noting that a certain generic drug name may have a different ‘brand name’. The prices of brand names differ enormously. That is the root reason for physicians’ profit-seeking behavior regarding prescriptions. The ‘4+7’ policy regulates prescription behavior and saves the expenditure of drugs by giving priority to cheaper bid-winning drugs. However, original drugs are still popular among patients because of their satisfactory therapeutic effects. Due to the preference for original drugs among physicians and patients and the agreed volume of bid-winning drugs, it is essential to explore the differences in the impact of the ‘4+7’ policy on the use of original and generic drugs.

### 1.3. City-Level and County-Level Hospitals

Nowadays, despite stable economic growth, accelerated city development, and the improved health status of residents in both rural and urban areas in China, there is still a noticeable urban–rural gap in health-related issues. City-level hospitals take on more of the quantity of healthcare services compared to county-level hospitals, which is influenced by multiple factors. The first is the imbalance in the allocation of healthcare resources. Hospitals in county areas own fewer numbers of healthcare facilities, available beds, health workforces, and health technologies [18], which results in the poor quality of healthcare services in county-level hospitals and patients’ preference for city-level hospitals. The second comprises the disparities in healthcare utilization behavior between urban and rural residents. Healthcare utilization behavior is attributed to predisposing (i.e., age, gender, family size, education, and health beliefs), enabling (i.e., health insurance, income, proximity, and availability and accessibility of healthcare services), and need (i.e., evaluated health status and perceived need) factors [19,20]. Compared with their urban counterparts, there are less opportunities for rural residents to use healthcare services, due to their lower attainment of education and income, their higher prevalence of unemployment, and the lower accessibility of health insurance and healthcare services for them [21,22]. Thus, there is less healthcare service utilization in county-level hospitals where the rural population is the majority. The third is because rural populations are in better health than urban populations [21]. Urban patients experience a higher incidence of breast cancer [23] and a higher prevalence of chronic diseases [24] and mental disorders [25], which could be attributed to urbanization. Hence, the healthcare utilization in county-level hospitals is naturally lower. Based on the difference in the quantity of healthcare utilization between city-level and county-level hospitals, it is necessary to examine city-county level hospitals’ disparity in the impact of the ‘4+7’ policy on the use of drugs.

### 1.4. Literature Review Regarding the Impact of ‘4+7’ Volume-Based Drug Procurement on the Use of Drugs

Under the ‘4+7’ policy, prescription priority should be given to bid-winning drugs. Wang et al. [26] found that after the ‘4+7’ policy in mainland China, the use of bid-winning original and generic drugs and ‘4+7’ policy-list generic drugs and policy-related generic drugs significantly increased, while the use of non-winning original and generic drugs and ‘4+7’ policy-list original drugs and policy-related original drugs significantly declined. Yang et al.’s study [27] in Shenzhen city indicated that the use of bid-winning antihypertensive drugs significantly increased and non-winning antihypertensive drugs significantly decreased after the ‘4+7’ policy. Chen et al.’s case study [28] in Shenzhen showed that the use of ‘4+7’ policy-related drugs increased by 73.8%, among which bid-winning drugs jumped by 1638.2% and non-winning drugs dropped by 70.8%. Using the Centralized Drug Procurement Survey in Shenzhen, Yang et al. [29] found the volume of cefuroxime and its alternative drugs increased by 92.9% and 30.2%, respectively after the ‘4+7’ policy.

Centralized drug procurement has been proven to save drug costs around the world. A study in Colombia showed that the centralized purchasing strategy saved the cost of drugs for hepatitis C and also procured savings for the healthcare system [30]. Another study in Italy indicated that the centralized pharmaceutical procurement system paid lower drug prices and the average cost saving was about 20% for agencies [31]. The pooled procurement system in India was estimated to save about 30% of the annual drug bill of the government [32]. The same scenario was seen in Greece [33] and Jordan [34]. In China, Wang et al. [26] found a prominent reduction in the monthly expenditure of non-winning drugs (CNY 771.37 million), ‘4+7’ policy-list drugs (CNY 726.40 million), and policy-related drugs (CNY 654.47 million) after the ‘4+7’ policy using eleven cities’ drug purchasing data. Using drug purchasing data in Shenzhen, Yang et al. [27] observed that the expenditure of non-winning, ‘4+7’ policy-list, and overall policy-related antihypertensive drugs significantly decreased by CNY 6.92, 5.96, and 8.02 million after the ‘4+7’ policy.

According to the above literature review, we find an increase in the volume of bid-winning, policy-list, and policy-related drugs, and a decline in the volume and the expenditure of non-winning, policy-list, and policy-related drugs after the ‘4+7’ policy in China. There is also prominent drug cost-saving success abroad with the implementation of centralized drug procurement. However, there is little discussion about the volume and the expenditure of original and generic drugs in different healthcare institutions. Given the priority of bid-winning generic drugs under ‘4+7’ policy rules, doctors’ profit-seeking motives for high-profit original drugs, and patients’ preference for original drugs, as well as the gaps in the quantity of healthcare utilization between city-level and county-level hospitals, the change in the use of drugs by the types of drugs and healthcare institutions is still unclear under the ‘4+7’ policy. Hence, it is of great significance to capture the differential impacts of the 4+7 policy on the use of original and generic drugs in city-level and county-level hospitals.

### 1.5. Purpose and Hypotheses of This Study

Shaanxi, located in northwestern China, is regarded as a northwest medical center and a major pilot province of northwestern health system reforms. Identifying the implementation effects of the ‘4+7’ policy in Shaanxi is crucial to understand the progress of CVBDP in northwestern China. Thus, taking Shaanxi as the research site, we conducted this natural experimental study to quantitatively assess the impact of the ‘4+7’ policy on the use of drugs and examine the heterogeneous impact of the ‘4+7’ policy across the types of drugs (i.e., original and generic drugs) and hospitals (i.e., city-level and county-level hospitals). We attempted to recognize the intended and unintended impacts of the ‘4+7’ policy on drug use and to provide empirical evidence for policymakers to set priorities and strategies.

According to the general market laws, when the use of one drug increases, its substitutable product will decrease. Under the ripple effect theory, the sharp decline in the price of bid-winning drugs will trigger a ‘domino effect’ in the pharmaceutical industry, which will then drive down the price of non-winning, alternative, policy-list, and policy-related drugs. Taking into account the combination of the volume and price of drugs, medication preference and the agreed volume of bid-winning drugs, as well as the difference in the quantity of healthcare utilization between city-level and county-level hospitals, we hypothesize that after the ‘4+7’ policy (1) the volume of bid-winning, policy-list, and policy-related drugs increases, while that of non-winning drugs and alternative drugs decreases; (2) the expenditure of bid-winning drugs increases, while that of non-winning, alternative, policy-list, and policy-related drugs decreases; (3) the volume and the expenditure of generic drugs change more than those of their original drug counterparts; and (4) the changes in the volume and the expenditure of drugs are greater in city-level hospitals compared with in county-level hospitals.

## 2. Methods

### 2.1. Study Setting

In this study, Shaanxi Province was selected as the research setting. It consists of 11 cities and 71 counties, with a 2019 population of 38.76 million and an area of 205,600 square kilometers [35]. In 2019, Shaanxi had 35,404 healthcare institutions, including 1209 hospitals, with an average of 2.8 physicians, 3.88 nurses, and 6.86 beds per 1000 residents [36].

### 2.2. Data Source

The data used in this study came from the Shaanxi Drug and Apparatus Centralized Procurement Platform (SDACPP), which covers all provincial drug and apparatus purchasing records from healthcare institutions in Shaanxi Province. The SDACPP integrates bidding, procurement, transactions, and supervisions. In the drug procurement block of the SDACPP database, each drug procurement order record includes the code of the drug, generic name, dosage form, specification parameter, conversion coefficient, purchase volume, purchase expenditure, pharmaceutical manufacturer, type of healthcare institution, and purchase date, etc.

### 2.3. Sample Selection

**Figure 1:**
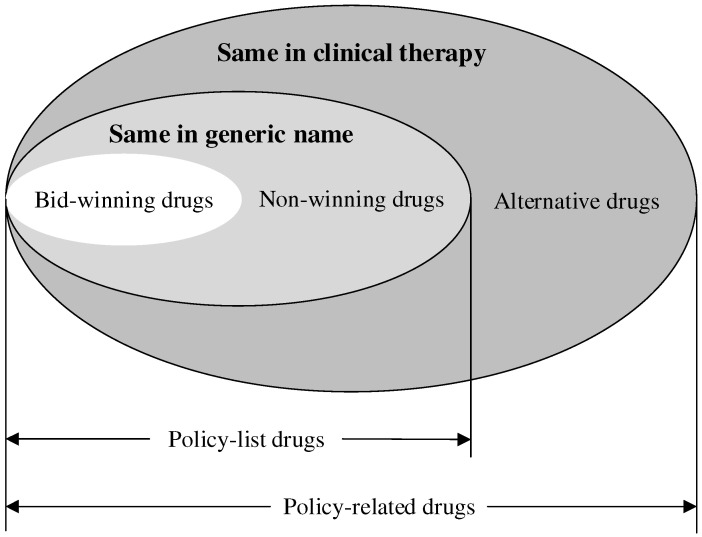
The relationship between each type of drug.

(1) The time period: The ‘4+7’ policy was implemented at the end of March 2019 in Xi’an, Shaanxi. Taking the influence of two additional policies into consideration, the Medical Service Price Reform in Shaanxi Province (April 2017), and the first-round CVBDP in Shaanxi Province (December 2019), this study delimited the time period from April 2017 to November 2019. Thus, 32 months were involved.
(2) The scope of cities: according to the drug procurement order records in the SDACPP, this study contained 12 cities: Xi’an, Xianyang, Shangluo, Ankang, Baoji, Yanan, Yangling, Yulin, Hanzhong, Weinan, Tongchuan, and Hancheng. Xi’an was the pilot city.
(3) The scope of healthcare institutions: we divided hospitals into city-level and county-level according to the SDACPP.
(4) The scope of drugs: Referring to previous studies [26,27,37], the ‘4+7’ policy-related drugs were analyzed in this study, containing the ‘4+7’ policy-list drugs and their alternative drugs. The ‘4+7’ policy-list drugs were 25 drugs (by generic name) in the ‘4+7’ procurement list, which were further divided into bid-winning drugs and non-winning drugs. Bid-winning drugs were products that won the tender, which were listed in the ‘4+7’ procurement bid-winning results [38]. Meanwhile, non-winning drugs were those with the same generic name but which lost the tender. The alternative drugs referred to the drugs that are clinically substitutable with the ‘4+7’ policy-list drugs, which were selected according to an official file [39]. Figure 1 indicates the relationship between each type of drug. All the drugs included in this study are listed in Table S1. In addition, all the drugs were classified into original and generic drugs.

Eventually, 111,999 drug procurement order records of 118 drugs (with generic names) were extracted in this study, including 31,310 orders of 25 ‘4+7’ policy-list drugs (including 6895 drug procurement order records of 25 bid-winning drugs and 24,415 drug procurement order records of 25 non-winning drugs) and 80,689 orders of 93 alternative drugs.

### 2.4. Outcome Indicators

The volume and the expenditure of drug procurement were selected as the outcome variables in this study. To ensure the comparability of drug use between different packages of medicines, Defined Daily Doses (DDDs), instead of the physical quantity, were used to measure the volume [40]. The DDD is a standard measurement developed by the World Health Organization (WHO) to calculate and compare drug consumptions, which is defined as the assumed average maintenance dose per day for a drug used for its main indication in adults [41]. The DDD value of each medication was determined in accordance with the Guidelines for Anatomical Therapeutic Chemical (ATC) classification and DDD assignment 2022 [42], *Newly Pharmacology* (17th Ed.) [43], and Medicine Specification. The DDD equivalence per package (DPP) of drugs is calculated using the formula as follows [12,29]:

(1) $$DPP=\frac{format\times size}{DDD}$$

where format refers to the conversion coefficient, and size refers to the specification parameter.

The volume for each procured drug with the same generic name, also known as DDDs, was calculated as the summed DPP of all the packaged products. Higher DDD values indicated that more drugs were used. DDDs are estimated using the formula as follows:

(2) $$DDDs=\sum_{i=1}^{n}{DPP}_{i}\times N_{i}$$

where $N_{i}$ refers to the number of packages of a certain product (i) purchased by the hospitals.

The expenditure of drugs was measured by the amount of drug purchase order records, calculated by Chinese yuan (CNY).

### 2.5. Explanatory and Control Variables

The key explanatory variable in this study was the ‘4+7’ policy intervention. It was an interaction term between the time period and the treatment/control group, with a value of 1 meaning that the treatment group was in the post-‘4+7’ policy intervention and a value of 0 indicating otherwise.

Due to the lack of hospital names in the drug procurement order records from the SDACPP database, we could not match the detailed hospital characteristics. However, several potential confounding factors were controlled in this study, such as the type of hospital (i.e., city/county-level of hospital), the type of drugs (i.e., original/generic drugs), the economic development at a city level (i.e., Gross Domestic Product per capita), and the healthcare resources at the city level (i.e., number of healthcare institutions per 10,000 population, number of beds per 1000 population, number of healthcare technical personnel per 1000 population).

### 2.6. Statistical Analysis

Descriptive statistical analysis was first employed. The change in the volume and the expenditure of the included drugs were described in the same period before (from March 2018 to November 2018) and after (from March 2019 to November 2019) the ‘4+7’ policy. Then, a difference-in-differences (DID) approach controlling for the potential confounders was applied to estimate the impact of the ‘4+7’ policy on the volume and the expenditure of drugs in different hospitals. The DID method has become an increasingly popular method to identify causal effects (i.e., the impact of a policy change) by contrasting the change in outcomes pre- and post-intervention for the treatment and control group [44]. The DID approach can detect the net impacts of the policy intervention on outcomes by separating the inherent difference between the treatment and control group from the total difference pre- and post-intervention. In this natural experimental study, we constructed DID model using the panel data, which is expressed as follows:

(3) $$Y_{it}=\beta_{0}+\beta_{1}{Treat}_{i}+\beta_{2}{Time}_{t}+\beta_{3}{Treat}_{i}\times {Time}_{t}+\gamma C_{it}+\mu_{i}{+\mu_{t}+\varepsilon }_{it}$$

where i and t represent the hospital attribute and time, respectively. $Y_{it}$ refers to the volume and the expenditure, which are the outcome indicators in this study. ${Treat}_{i}$ refers to the group, where the values of 0 and 1 represent the control group (hospitals except for those located in Xi’an city) and treatment group (hospitals located in Xi’an city), respectively.$\;{Time}_{t}$ refers to the period, where the values of 0 and 1 represent the pre-‘4+7’ policy (from April 2017 to February 2019) and the post-‘4+7’ policy (from March 2019 to November 2019), respectively. $C_{it}$ refers to the control variables. $\mu_{i}$ and $\mu_{t}$ represent the hospital attribute fixed effect and month fixed effect, respectively. Since the exact hospital name could not be identified in the SDACPP, a hospital attribute was employed instead of an individual fixed effect. There were 13 hospital attributes in total, including 1 provincial and 12 municipal hospitals (same as the city name). $\varepsilon_{it}$ is the error term. $\beta_{3}$, the key parameter of interest, measures the net impacts of the ‘4+7’ policy on the outcome indicators. Table 1 presents the explanation of the parameters of DID.

The DID design relies on the assumption that, in the absence of an intervention, both the treatment group and the control group would have followed the same temporal trends in outcomes on average over time [45]. This assumption is known as the ‘parallel trends assumption’, which allows the differences in the averages of the unobserved variables between the treatment and the control group, but these differences do not change over time [46]. We tested the parallel trends using an event study according to the previous literature [47,48].

Furthermore, placebo analysis is essential to ensure that the impacts measured can be attributed to the introduction of the ‘4+7’ policy rather than to other unobserved factors. It typically comes in three forms, that is, the in-time placebo test [49], the in-space placebo test [50], and the mixed placebo test [51]. The in-time placebo test makes the use of a fake treatment time before the treatment actually starts, which is also called ‘backdating’. We assumed the fake treatment time was March 2018, referring to previous studies [52]. The in-space placebo test uses the fake treatment units for statistical inference. We selected Shangluo city as a fake pilot city since it differs in healthcare and economic development from Xi’an city, so as to avoid contamination. Hospitals except for those located in Xi’an city were considered as the control unities. The mixed placebo test uses a fake treatment time and fake treatment units simultaneously; that is, it applies the in-space placebo test with a fake treatment time. We regarded hospitals in Shangluo city as the fake treatment units and those in cities except for Xi’an as the control unities and March 2018 as the fake treatment time. The insignificant estimation of the placebo analysis would address the concern as to whether there was another exogenous event or omitted variables so that the impacts could be ascribed to the ‘4+7’ policy.

Two fixed-effect models were employed in the whole analyses. Model 1 was a crude analysis without controlling for any confounders, and Model 2 adjusted for the various confounding factors mentioned. Stata software (version 15.0; Stata Corp, College Station, TX, USA) was used to perform the analyses above. All statistical tests were two-sided, with a significance threshold of 0.05.

## 3. Results

### 3.1. The Change in the Use of Drugs in the Pilot City Pre- and Post-‘4+7’ Policy

Table 2 presents the overall change in the use of drugs in the pilot city. The volume of the policy-list drugs, bid-winning drugs, alternative drugs, and policy-related drugs increased by 142.11%, 588.97%, 26.96%, and 68.16%, respectively, while that of the non-winning drugs decreased by 32.06% after the ‘4+7’ policy. Regarding the expenditure after the ‘4+7’ policy, declines of 21.80%, 43.36%, and 0.67% for the policy-list drugs, non-winning drugs, and policy-related drugs were observed, and increases of 24.99% and 23.89% for the bid-winning and alternative drugs were detected.

Table 3 presents the change in the use of drugs by the types of drugs and hospitals in the pilot city. The volume and the expenditure of the non-winning original and generic drugs decreased, whereas that of the alternative original and generic drugs increased. Compared with city-level hospitals, there was a larger change in the volume and the expenditure of the policy-list drugs, bid-winning drugs, and non-winning drugs in county-level hospitals. However, the volume and the expenditure of the alternative drugs in city-level hospitals increased more than those in county-level hospitals. The detailed change in the use of original and generic drugs in different hospitals in the pilot city is presented in Table S2.

The change in the volume proportion of the policy-list and policy-related generic drugs in the pilot city is presented in Table 4. After the ‘4+7’ policy, 25.02% of policy-list original drugs were replaced by their generic product substitutions in the pilot city. The policy-list and policy-related generic drug substitution was higher in city-level hospitals compared with in county-level hospitals (11.21% vs. 26.85% among policy-list drugs, 16.73% vs. 9.13% among policy-related drugs). In addition, a higher use proportion of generic drugs in county-level hospitals and a higher growth rate of generic drug substitution in city-level hospitals were found.

### 3.2. The Impact of the ‘4+7’ Policy on the Use of Drugs

Table 5 shows the DID estimates of the impact of the ‘4+7’ policy on the use of overall drugs. In Model 2, adjusting the confounding factors and the volume of policy-list (Coef. = 204.76, *p* < 0.001), bid-winning (Coef. = 256.72, *p* < 0.01), alternative (Coef. = 44.05, *p* < 0.05), and policy-related drugs (Coef. = 250.97, *p* < 0.001) significantly increased after the ‘4+7’ policy. The expenditure of policy-list (Coef. = −414.11, *p* < 0.01) and non-winning (Coef. = −551.47, *p* < 0.01) drugs significantly decreased, and that of bid-winning (Coef. = 140.11, *p* < 0.01) and alternative (Coef. = 181.87, *p* < 0.05) drugs significantly increased after the ‘4+7’ policy.

The DID results of the impact of the ‘4+7’ policy on the use of drugs by the types of drugs and hospitals are presented in Tables S3 and S4, respectively. Table 6 shows the results of the impact of the ‘4+7’ policy on the volume of original and generic drugs in different hospitals. After the ‘4+7’ policy, the volume of policy-list generic drugs in county-level and city-level hospitals, as well as all kinds of bid-winning drugs except for original drugs with no records, increased after the ‘4+7’ policy. The decline in the volume of non-winning original and generic drugs was only found in county-level hospitals. In addition, we found the volume of alternative original drugs in city-level hospitals increased 38.36 ten thousand DDD (Coef. = 38.36, *p* < 0.001) after the ‘4+7’ policy. The DID estimates of the impact of the ‘4+7’ policy on the expenditure are listed in Table S5.

### 3.3. Parallel Trend Test and Placebo Test

**Figure 2:**
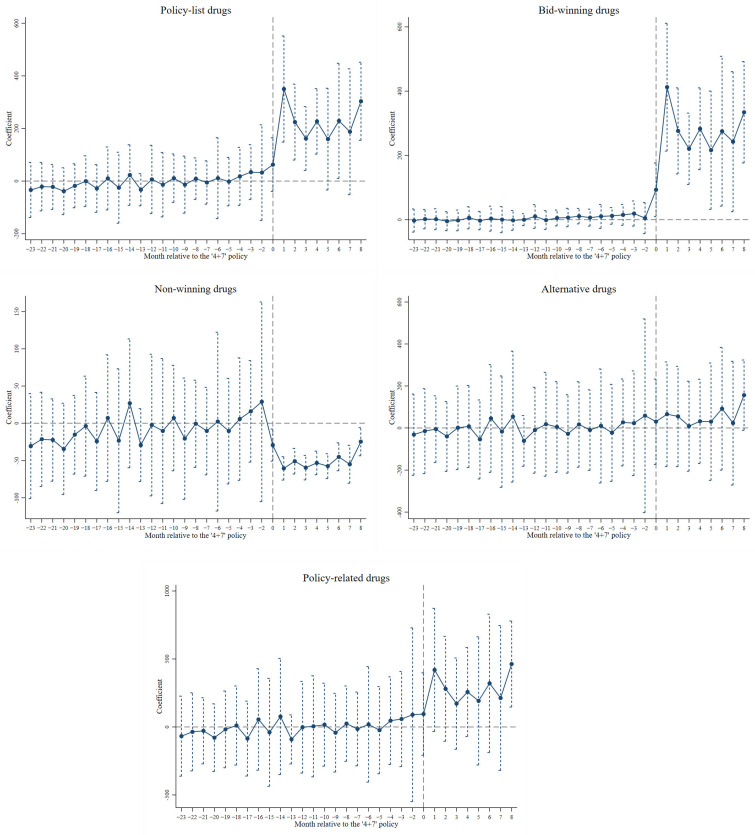
Parallel trend test on the impact of the ‘4+7’ policy on the volume of drugs.

Figure 2 and Figure S1 present the results of the parallel trend test on the impact of the ‘4+7’ policy on the volume and the expenditure of drugs, respectively. The insignificant estimates before the ‘4+7’ policy demonstrated that the treatment and the control group followed the same temporal trends in outcomes on average over time, indicating that the differences in the averages of the unobserved variables remained unchanged between the treatment and the control group.

To further ensure that the DID estimates of the impact of the ‘4+7’ policy was not affected by other unobservable policies or factors, we conducted the in-time placebo test, the in-space placebo test, and the mixed placebo test. As shown in Tables S6–S8, the majority of the insignificant estimates in Model 2 addressed the change in the use of drugs, which could be ascribed to the ‘4+7’ policy rather than to other unmeasured factors (i.e., the systematic difference among hospitals).

## 4. Discussion

This study attempts to make contributions to a full understanding of the impact of CVBDP, specifically the ‘4+7’ policy, on the use of original and generic drugs in county-level and city-level hospitals by using a DID approach over a 23-month period in Shaanxi, China. The heterogeneous effects of the ‘4+7’ policy on drug use by the types of drugs and hospitals was examined. The key findings are as follows: after the ‘4+7’ policy, (1) the volume of policy-list, bid-winning, and policy-related drugs increased; (2) the expenditure of policy-list and non-winning drugs decreased; (3) bid-winning and policy-related generic drugs increased more in volume than their original drug counterparts, and the expenditure of non-winning generic drugs reduced more than that of non-winning original drugs; (4) the volume and the expenditure of policy-list, bid-winning, and alternative drugs in city-level hospitals changed more than those in county-level hospitals; (5) generic drug substitution was stronger in city-level hospitals, and the volume and the expenditure of alternative drugs was unexpectedly increased, especially the volume of alternative original drugs in city-level hospitals.

It was found that the volume of policy-list, bid-winning, and policy-related drugs increased after the ‘4+7’ policy, with growth rates of 142.11%, 588.97%, and 68.16%, respectively. The increase in the volume of bid-winning drugs is in line with the findings of antihypertensive drugs in Yang et al.’s study [27] and overall drugs in Chen et al.’s study [28]. On the one hand, it is likely that the released medication demand, which was hidden before the ‘4+7’ policy [29], can be considered as demonstrating improved access to medicine. On the other hand, it could be attributed to the increasing course of treatment or single doses, under the requirement for the agreed procurement volume of the selected products. It is worth noting that physicians may appear to prescribe the bid-winning drugs in a ‘one size fits all’ manner in order to fulfill the political task of the agreed procurement volume. In addition, a prominent reduction was observed in the monthly expenditure of overall policy-list drugs and non-winning drugs, consistent with previous studies [26,27]. Similar findings were found in other countries. A study in Mexico indicated that the Price Negotiation for 12 antiretroviral drugs reduced total spending by 38% [53]. The decrease in the spending of the overall 25 policy-list drugs confirmed the success in pharmaceutical cost containment during the process of the ‘4+7’ policy. The linkage of volume and price plays a key role in the shaping of the mechanism of pharmaceutical price and cost saving regarding pharmaceutical and medical insurance funds.

In terms of the heterogeneity analyses by drug type, we found that the volume of bid-winning and policy-related generic drugs increased more than that of original drugs. Similar results were also observed in Wang et al.’s study [26] and Duan et al.’ s study [54]. After the ‘4+7’ policy, it was indicated 25.02% and 15.30% of generic drugs were substituted for original ones among policy-list drugs and policy-related drugs, respectively. A similar finding was also detected in a previous study [26], indicating the expansion of the generic pharmaceutical market and the increasing generic drug substitution. We also observed a higher use proportion of generic drugs in county-level hospitals, and a higher growth rate of generic drug substitution in city-level hospitals. This presents a higher baseline of generic drug prescriptions in county-level hospitals and an accelerated process of generic drug substitution in city-level hospitals. Furthermore, we found that the expenditure of both non-winning original and generic drugs reduced after the ‘4+7’ policy. Compared to their original counterparts, non-winning generic drug products decreased more. We suppose this is the consequence of the competition for limited pharmaceutical market shares between non-winning and bid-winning drug products and the unprecedented profit squeeze because of the ‘4+7’ policy, especially among generic drug manufacturers. With regard to the heterogeneity analyses by hospital type, they presented greater changes in the volume and the expenditure of policy-list, bid-winning, alternative, and policy-related drugs in city-level hospitals, indicating better policy execution and impacts in city-level hospitals, also known as large healthcare institutions [26]. Compared to county-level hospitals, the demand and utilization of healthcare services and medicines are greater in city-level hospitals.

This raises our vigilance given that the volume and the expenditure of alternative drugs are consistent with the results of antibiotics in Yang et al.’s study in Shenzhen city [29] and those of antineoplastic drugs in Duan et al.’s national study [54]. The increase in the expenditure of alternative drugs could be ascribed to increased volume. It might also be associated with pharmaceutical manufacturers’ cost-shifting behaviors, as profit-driven pharmaceutical manufacturers are likely to compensate for the squeezed profit margins derived from bid-winning drugs. Specifically, the incremental volume of alternative drugs was embodied in original drugs in city-level hospitals. As for how no such finding was found among alternative drugs in county-level hospitals, this might be related to the preference for original drugs in city-level hospitals and lower acceptability for bid-winning drugs among patients in urban areas [55]. The unanticipatedly incremental volume of alternative original drugs could be explained by the poor clinical efficacy of bid-winning drugs. Only generic drugs that have previously passed the GCE and original drugs are eligible to enter into CVBDP. There are three levels of equivalence tests: a pharmaceutical equivalence test, a bioequivalence test, and a therapeutic equivalence test. In China, the pharmaceutical equivalence test and bioequivalence test are the main components for the GCE. The therapeutic equivalence test relies on large-scale clinical data in the real world [56]. Evidence shows that the clinical therapeutic efficacy of some bid-winning drugs is indeed unsatisfactory. The side effects of the selected drugs increase, and even a double dose of the selected drugs are not as good as a standard dose of the original drugs, for instance, Atorvastatin, Amlodipine Besylate, Losartan, Glimepiride, and Azithromycin [57]. A systematic review also indicated that 23.6% pharmacists, 28.7% doctors, and 35.6% lay people viewed generics as less effective than branded drugs [58].

Poor acceptability and compliance with bid-winning drugs among patients could also be one possible reason for the increase in the volume of alternative original drugs. It might be attributed to the change in patients’ usual medication habits and doubts about the quality of cheaper generic drugs. Evidence shows that 35.6%, 18.0%, and 18.8% of residents hold negative perceptions of generics, had more safety concerns about generics, and believed generics caused more side effects than branded drugs, respectively [58]. A survey also indicated that generic labels negatively affected adherence to treatment even if patients report ex ante positive evaluations of the quality of generic drugs [59].

In addition, given that physicians still have profit-seeking behaviors towards those drugs with equal clinical efficacy to bid-winning drugs but which are more expensive, alternative drugs are more likely to be prescribed, especially original drug products. China has a healthcare system in which physicians can both prescribe and dispense drugs. The healthcare insurance system provides universal coverage for prescription drugs in the National Drug Reimbursement List (NDRL), which results in the government regulating the reimbursement (retail) price but not regulating the acquisition (wholesale) price of the pharmaceuticals. Physicians gain profits directly from the sale of prescription drugs unlisted in the NDRL or those with a large profit margin between the regulated reimbursement price and the unregulated acquisition price [60]. When it comes to CVBDP, physicians under financial incentives are at risk of prescribing unregulated substitutable original drugs. The government needs to take physicians’ financial incentives into consideration when setting the reimbursement price for original and generic drugs, to progressively enhance generic drug competition and substitution. It is worth noting that the potential reasons discussed above for the incremental volume of alternative original drugs could be more convincing when the quality and the therapeutic effect of bid-winning drugs, the perceptions of generic drugs, the medication preference of patients, and a prescriber’s motivation are quantificationally measured.

Volume-based drug procurement is considered as a common practice to lower pharmaceutical expenditure globally [10]. The decline in the expenditures of the selected and the overall medicines were also observed in South America, Europe, and Asia, such as in Colombia [30], Italy [31], Greece [33], India [32], and Jordan [34]. According to the general laws of the market, the concerns about the safety of generic drugs, the preference for original drugs among physicians and patients, and doctors’ profit-seeking motivations, as well as the uneven development of healthcare institutions in urban and rural areas, we suppose that similar findings regarding the change in the volume could be found in other countries.

The current study supplements and improves upon the existing literature on the impact of the ‘4+7’ policy on the use of drugs and provides empirical evidence on identifying the differential effects of the ‘4+7’ policy on drug use across original–generic products and urban–rural healthcare institutions. The findings not only verify the success in generic drug substitution and the pharmaceutical expenditure containment under the background of the ‘4+7’ policy but also reveal the incremental volume and expenditure of alternative drugs, especially the increase in the volume of alternative original drugs in city-level hospitals, suggesting the potential risk of the poor quality of bid-winning drugs, lower acceptability of bid-winning drugs among residents, and irrational use of drugs under physicians’ profit-seeking behaviors. The findings may assist health policymakers in setting priorities and in improving supporting measures and policies. To be specific, given that the indications of policy-related drugs are highly consistent, the selection of bid-winning drugs should be guided by clinical need and give priority to diseases with relatively fewer alternative drugs, such as ophthalmology and mental disorders. Brand-name preference among prescribers and patients is regarded as a key influencing factor of generic drug substitution. Thus, generic drugs with fewer alternative original drugs are better to be listed as selected drugs. In order to reduce the safety concerns about generic drugs, much supervision and regulation are needed to be strengthened to ensure the quality of generic drugs at both the government level and pharmaceutical manufacturer level. A therapeutic equivalence test should be considered to add into the GCE if possible. Furthermore, it is vital for the National Health Commission to take measures to improve the progress of CVBDP in county-level hospitals, so as to achieve universal generic drug substitution and pharmaceutical cost saving in the health system.

We acknowledged several limitations regarding this study. Firstly, given that a prescription is probably affected by a patient’s condition, medical history, medication habits, and affordability, the alternative drugs included in this study may not be comprehensive in covering all drugs that are clinically equal to bid-winning drugs. Secondly, drug procurement data instead of prescription data were applied in this study, as there is strong concordance between drug purchases and drug prescriptions. However, this might result in an incomplete measurement of drug use, for instance, the dosage, duration, and joint use of selected drugs. It is essential to clarify the change in drug use using detailed prescription information in the future. Thirdly, several confounding factors (i.e., types of hospitals and drugs, economic development, and healthcare resources at the city level), hospital attribute fixed effects, and time fixed effects were controlled, and a parallel trend test and three placebo tests were adopted in analyses, which make the findings credible. However, we cannot deny that the results might be biased due to the lack of the physician’s characteristics (i.e., age, gender, education, economic level, years of working) and patient information regarding social–economic characteristics, disease type, and medication preference, as well as the hospital characteristics (i.e., name, number of outpatients and inpatients). It is valuable for future investigations using more comprehensive data to address this. Fourthly, considering the quantity of healthcare service utilization, primary healthcare settings were not involved in this study. Based on the larger proportion of the essential drugs in primary healthcare settings, it is of great necessity to explore the impact of CVBDP on the use of policy-related drugs in community health service centers and township health centers in future. Fifthly, we cannot distinguish public and private hospitals from the overall hospitals because of the limitation of the database. Under the several rounds of CVBDP, it is essential to examine the change in the use of drugs in private healthcare institutions (i.e., private hospitals and retail pharmacies) further to make contributions to the construction of a healthy, ordered, and competitive health system. Finally, the present study examined the impact of the ‘4+7’ policy in Shaanxi Province; thus, the findings may not generalize to CVBDP nationwide. It is necessary to evaluate more rounds of CVBDP on the use of drugs, the quality of bid-winning drugs, patients’ satisfaction and health outcomes, as well as the landscape of pharmaceutical manufacturers and pharmaceutical distribution companies, by using a nationwide database.

## 5. Conclusions

The present study highlights the evident generic drug substitution and pharmaceutical expenditure saving after the ‘4+7’ policy, especially in city-level hospitals. Meanwhile, the unanticipatedly increasing volume and expenditure of alternative drugs, especially the incremental volume of original drugs in city-level hospitals, suggests the potential risk of the poor quality of bid-winning drugs, lower compliance with bid-winning drugs among patients, or physicians’ profit-seeking behaviors. More regulations and supervisions for the prescription and financial incentives of physicians are needed to address these concerns.

## Figures and Tables

**Table 1:** The explanation of parameters of DID.

Group	Pre-	Post-	Post-Minus Pre-(Net Effect)
Control group	*β*_0_	*β*_0_ + *β*_2_	*β*_2_
Treatment group	*β*_0_ + *β*_1_	*β*_0_ + *β*_1_ + *β*_2_ + *β*_3_	*β*_2_ + *β*_3_
Treatment group–control group	*β*_1_	*β*_1_ + *β*_3_	*β*_3_

**Table 2:** The overall change in the use of drugs in the pilot city.

Variables	Volume (Ten Thousand DDDs)			Expenditure (Ten Thousand CNY)		
	Pre-	Post-	GR (%)	Pre-	Post-	GR (%)
Policy-list drugs	2855.98	6914.72	142.11	29,783.08	23,291.81	−21.80
Bid-winning drugs	800.98	5518.49	588.97	9395.72	11,743.49	24.99
Non-winning drugs	2054.99	1396.23	−32.06	20,387.36	11,548.32	−43.36
Alternative drugs	5126.65	6508.79	26.96	25,612.09	31,730.16	23.89
Policy-related drugs	7982.63	13,423.51	68.16	55,395.17	55,021.97	−0.67

Note: DDD: Defined Daily Dose; CNY, Chinese yuan; Pre-: March 2018 to November 2018; Post-: March 2019 to November 2019; GR: growth rate.

**Table 3:** The change in the use of drugs by types of drugs and hospitals in the pilot city.

Variables	Volume (Ten Thousand DDDs)			Expenditure (Ten Thousand CNY)		
	Pre-	Post-	GR (%)	Pre-	Post-	GR (%)
**Difference in drugs**						
Policy-list drugs						
Original	1146.32	1045.83	−8.77	11,019.16	7652.33	−30.55
Generic	1709.66	5868.89	243.28	18,763.92	15,639.48	−16.65
Bid-winning drugs						
Original	2.53	28.57	1029.25	11.88	39.52	232.66
Generic	798.45	5489.93	587.57	9383.84	11,703.97	24.72
Non-winning drugs						
Original	1143.79	1017.26	−11.06	11,007.28	7612.81	−30.84
Generic	911.21	378.96	−58.41	9380.08	3935.50	−58.04
Alternative drugs						
Original	3007.24	3884.51	29.17	7023.01	10,983.38	56.39
Generic	2119.41	2624.28	23.82	18,589.08	20,746.78	11.61
Policy-related drugs						
Original	4153.56	4930.34	18.70	18,042.17	18,635.71	3.29
Generic	3829.07	8493.17	121.81	37,353.00	36,386.26	−2.59
**Difference in hospitals**						
Policy-list drugs						
County-level	378.23	1043.38	175.86	2506.32	1567.46	−37.46
City-level	2477.74	5871.34	136.96	27,276.75	21,724.34	−20.36
Bid-winning drugs						
County-level	92.63	979.86	957.82	626.56	1068.78	70.58
City-level	708.35	4538.63	540.73	8769.16	10,674.71	21.73
Non-winning drugs						
County-level	285.60	63.51	−77.76	1879.76	498.69	−73.47
City-level	1769.39	1332.71	−24.68	18,507.60	11,049.63	−40.30
Alternative drugs						
County-level	1002.84	1102.50	9.94	2720.05	3100.91	14.00
City-level	4123.81	5406.29	31.10	22,892.05	28,629.26	25.06
Policy-related drugs						
County-level	1381.07	2145.88	55.38	5226.37	4668.37	−10.68
City-level	6601.55	11,277.64	70.83	50,168.80	50,353.60	0.37

Note: DDD: Defined Daily Dose; CNY, Chinese yuan; Pre-: March 2018 to November 2018; Post-: March 2019 to November 2019; GR: growth rate.

**Table 4:** The change in the volume proportion of policy-list and policy-related generic drugs in the pilot city (%).

Variables	Pre-	Post-	Diff	GR
Policy-list drugs				
Subtotal	59.86	84.88	25.02	41.78
County-level hospitals	84.50	95.71	11.21	13.26
City-level hospitals	56.10	82.95	26.85	47.86
Policy-related drugs				
Subtotal	47.97	63.27	15.30	31.90
County-level hospitals	61.43	70.56	9.13	14.86
City-level hospitals	45.15	61.88	16.73	37.06

Note: Pre-: March 2018 to November 2018; Post-: March 2019 to November 2019; Diff: the gap between the volume proportion of drugs from March 2018 to November 2018 and from March 2019 to November 2019; GR: growth rate.

**Table 5:** The impact of the ‘4+7’ policy on the use of overall drugs.

Variables	Volume (Ten Thousand DDDs)		Expenditure (Ten Thousand CNY)	
	Model 1	Model 2	Model 1	Model 2
**Policy-list drugs**				
Treat × Time	221.30 ***	204.76 ***	−319.95 *	−414.11 **
	(29.77)	(31.30)	(119.11)	(124.14)
Month fixed effect	Yes	Yes	Yes	Yes
Hospital attribute fixed effect	Yes	Yes	Yes	Yes
R^2^	0.789	0.805	0.335	0.385
**Bid-winning drugs**				
Treat × Time	262.95 **	256.72 **	173.81 ***	140.11 **
	(57.71)	(58.96)	(9.89)	(31.26)
Month fixed effect	Yes	Yes	Yes	Yes
Hospital attribute fixed effect	Yes	Yes	Yes	Yes
R^2^	0.827	0.830	0.472	0.523
**Non-winning drugs**				
Treat × Time	−41.87	−50.87	−494.56 **	−551.47 **
	(28.37)	(30.14)	(121.04)	(131.33)
Month fixed effect	Yes	Yes	Yes	Yes
Hospital attribute fixed effect	Yes	Yes	Yes	Yes
R^2^	0.386	0.426	0.485	0.509
**Alternative drugs**				
Treat × Time	64.49 ***	44.05 *	310.34 ***	181.87 *
	(12.58)	(15.11)	(42.57)	(79.67)
Month fixed effect	Yes	Yes	Yes	Yes
Hospital attribute fixed effect	Yes	Yes	Yes	Yes
R^2^	0.463	0.506	0.428	0.472
**Policy-related drugs**				
Treat × Time	285.79 ***	250.97 ***	−9.61	−229.90
	(41.60)	(44.85)	(151.71)	(213.13)
Month fixed effect	Yes	Yes	Yes	Yes
Hospital attribute fixed effect	Yes	Yes	Yes	Yes
R^2^	0.665	0.693	0.341	0.394

Note: DDD: Defined Daily Dose; CNY, Chinese yuan; Model 1: crude logistic regression; Model 2: adjusted logistic regression controlling the confounders. Robust standard errors are presented in parentheses. *** *p* < 0.001, ** *p* < 0.01, * *p* < 0.05.

**Table 6:** The impact of the ‘4+7’ policy on the volume of original and generic drugs in different hospitals.

Variables	County-Level Hospitals				City-Level Hospitals			
	Original		Generic		Original		Generic	
	Model 1	Model 2	Model 1	Model 2	Model 1	Model 2	Model 1	Model 2
**Policy-list drugs**								
Treat × Time	−4.94 **	−5.33 *	67.31 ***	61.11 ***	−2.58	−8.95	197.06 ***	188.44 ***
	(1.08)	(2.05)	(2.71)	(5.38)	(6.71)	(7.90)	(7.00)	(9.83)
Month fixed effect	Yes	Yes	Yes	Yes	Yes	Yes	Yes	Yes
Hospital attribute fixed effect	Yes	Yes	Yes	Yes	Yes	Yes	Yes	Yes
R^2^	0.375	0.422	0.736	0.759	0.155	0.206	0.854	0.859
**Bid-winning drugs**								
Treat × Time	/	/	96.39 ***	91.67 ***	1.37 **	1.02 *	214.38 ***	210.35 ***
	/	/	(1.26)	(2.66)	(0.24)	(0.34)	(19.40)	(10.87)
Month fixed effect	Yes	Yes	Yes	Yes	Yes	Yes	Yes	Yes
Hospital attribute fixed effect	Yes	Yes	Yes	Yes	Yes	Yes	Yes	Yes
R^2^	/	/	0.864	0.869	0.836	0.843	0.874	0.877
**Non-winning drugs**								
Treat × Time	−5.01 **	−5.40 *	−29.11 ***	−30.63 ***	−3.94	−10.22	−17.44	−22.01
	(1.08)	(2.03)	(1.55)	(2.89)	(6.90)	(8.08)	(12.43)	(12.26)
Month fixed effect	Yes	Yes	Yes	Yes	Yes	Yes	Yes	Yes
Hospital attribute fixed effect	Yes	Yes	Yes	Yes	Yes	Yes	Yes	Yes
R^2^	0.374	0.420	0.579	0.610	0.158	0.208	0.350	0.381
**Alternative drugs**								
Treat × Time	6.82 *	3.15	−15.90 **	−19.58 **	49.22 ***	38.36 ***	25.87 *	16.40 ^†^
	(2.61)	(4.20)	(4.05)	(5.15)	(3.92)	(5.13)	(10.88	(9.04)
Month fixed effect	Yes	Yes	Yes	Yes	Yes	Yes	Yes	Yes
Hospital attribute fixed effect	Yes	Yes	Yes	Yes	Yes	Yes	Yes	Yes
R^2^	0.391	0.400	0.461	0.486	0.383	0.411	0.341	0.387
**Policy-related drugs**								
Treat × Time	1.88	−2.17	51.41 ***	41.54 ***	46.64 **	29.47 *	222.93 ***	204.84 ***
	(3.36)	(3.67)	(4.97)	(6.49)	(10.64)	(12.27)	(17.84)	(18.60)
Month fixed effect	Yes	Yes	Yes	Yes	Yes	Yes	Yes	Yes
Hospital attribute fixed effect	Yes	Yes	Yes	Yes	Yes	Yes	Yes	Yes
R^2^	0.430	0.443	0.619	0.646	0.310	0.344	0.800	0.811

Note: Model 1: crude logistic regression; Model 2: adjusted logistic regression controlling the confounders. Robust standard errors are presented in parentheses. *** *p* < 0.001, ** *p* < 0.01, * *p* < 0.05, ^†^ *p* < 0.1.

## Data Availability

The datasets presented in this article are not readily available because they were used under license. Requests to access the datasets should be directed to the Health Commission of Shaanxi Province.

## Supplementary Materials

The following supporting information can be downloaded at https://www.mdpi.com/article/10.3390/healthcare13060686/s1, Table S1: The list of included drugs, Table S2: The change in the use of original and generic drugs in different hospitals in the pilot city, Table S3: The impact of the ‘4+7’ policy on the use of original and generic drugs, Table S4: The impact of the ‘4+7’ policy on the use of drugs in different hospitals, Table S5: The impact of the ‘4+7’ policy on the expenditure of original and generic drugs in different hospitals, Table S6: The in-time placebo test on the impact of the ‘4+7’ policy on the use of drugs, Table S7: The in-space placebo test on the impact of the ‘4+7’ policy on the use of drugs, Table S8: The mixed placebo test on the impact of the ‘4+7’ policy on the use of drugs, Figure S1: The parallel trend test on the impact of the ‘4+7’ policy on the expenditure of drugs.

## Abbreviations

OECD: Organization for Economic Cooperation and Development; CVBDP: Centralized Volume-Based Drug Procurement; PVA: price–volume agreement; NHSA: National Healthcare Security Administration; GCE: Generic Consistency Evaluation; SDACPP: Shaanxi Drug and Apparatus Centralized Procurement Platform; DDDs: Defined Daily Doses; ATC: Anatomical Therapeutic Chemical; WHO: World Health Organization; DPP: DDD equivalence per package; CNY: Chinese yuan; DID: difference-in-differences; NDRL: National Drug Reimbursement List.
