# Supplementary material for: Intended and Unintended Impacts of ‘4+7’ Volume-Based Drug Procurement on the Use of Drugs in China: A Natural Experimental Study
Source: Healthcare (Basel). 2025 Mar 20;13(6):686. doi: 10.3390/healthcare13060686 (PMC11941950; doi:10.3390/healthcare13060686)
Supplement: Supplementary file 1 [file healthcare-13-00686-s001.zip › healthcare-3502664-supplementary.pdf]

Intended and Unintended Impacts of '4+7' Volume-Based Drug Procurement on the Use of Drugs in China: A Natural Experimental Study

Supplementary material

Table S1 The list of included drugs

No.	'4+7' policy-list drugs	Alternative drugs
1	Atorvastatin	Pitavastatin, Fluvastatin, Lovastatin, Pravastatin, Simvastatin, Ezetimibe, Xuezhikang
2	Rosuvastatin	Pitavastatin, Fluvastatin, Lovastatin, Pravastatin,, Simvastatin, Ezetimibe, Xuezhikang
3	Clopidogrel	Aspirin, Tiolopidine, Ticagrelor, Cilostazol
4	Irbesartan	Captopril, Perindopril
5	Amlodipine Besylate	Felodipine, Nifedipine, Amlodipine and Atorvastatin, Amlodipine and Benazepril, Benidipine, Lercanidipine, Aspartic Acid Amlodipine, Nicardipine, Nitrendipine, Cilnidipine, Nifedipine, Valsartan and Amlodipine
6	Entecavir	Tenofovir Disoproxil, Lamivudine, Tenofovir disoproxil and efavirenz, Adefovir Dipivoxil
7	Escitalopram	Citalopram, Sertraline, Fluvoxamine, Bupropion Hydrochloride, Fluoxetine Hydrochloride, Trazodone Hydrochloride, Sertraline Hydrochloride, Venlafaxine Hydrochloride
8	Paroxetine	Paroxetine, Sertraline, Fluvoxamine, Fluoxetine, Sertraline, Venlafaxine, Clomipramine, Milnacipran, Trazodone
9	Olanzapine	Paliperidone, Clozapine, Aripiprazole, Metamizole and Chlorpromazine, Amisulpride, Loxapine, Haloperidol, Quetiapine, Fluphenazine Decanoate, Paliperidone, Chlorpromazine, Ziprasidone, Trifluoperazine, Trifluperidol, Ziprasidone
10	Cefuroxime	Cefalexin, Cefepime, Cefprozil, Cefdinir, Cefuroxime, Cefmenoxime, Cefaclor, Cefixime, Cefadroxil
11	Risperidone	Quetiapine, Aripiprazole, Metamizole and Chlorpromazine, Amisulpride, Loxapine, Perphenazine, Haloperidol, Droperidol, Fluphenazine Decanoate, Clozapine, Chlorprothixene, Paliperidone, Sulpiride, Penfluridol, Fluphenazine, Tiapride, Chlorpromazine, Chlorprothixene, Ziprasidone, Trifluoperazine, Ziprasidone, Pipotiazine, Paliperidone
12	Gefitinib	Afatinib, Osimertinib, Erlotinib, Icotinib
13	Fosinopril	Captopril, Perindopril

14	Irbesartan and Hydrochlorothiazide	Olmesartan and Hydrochlorothiazide, Losartan and Hydrochlorothiazide, Telmisartan and Hydrochlorothiazide, Valsartan and Hydrochlorothiazide, Allisartan Isoproxil, Olmesartan Medoxomil, Candesartan, Telmisartan, Valsartan, Eprosartan
15	Lisinopril	Captopril, Perindopril
16	Tenofovir Disoproxil	Lopinavir and ritonavir, Stavudine, lamivudine and nevirapine, Zidovudine and Lamivudine
17	Losartan	Candesartan, Candesartan Cilexetil, Telmisartan, Valsartan, Losartan and Hydrochlorothiazide, Telmisartan and amlodipine, Telmisartan and Hydrochlorothiazide, Valsartan and amlodipine, Valsartan and amlodipine, Valsartan and Hydrochlorothiazide
18	Enalapril	Captopril, Perindopril
19	Levetiracetam	Oxcarbazepine, Magnesium Valproate, Sodium Valproate, Carbamazepine, Lamotrigine, Topiramate
20	Imatinib	Dasatinib, Nilotinib
21	Montelukast	Pemirolast, Pranlukast, Seratrodast, Ibudilast, Zafirlukast
22	Diosmectite	Loperamide, Tannalbin Albumini
23	Pemetrexed	/
24	Flurbiprofen	Piroxicam, Diclofenac, Ketorolac Tromethamine, Indomethacin, Salicylic Acid
25	Dexmedetomidine	Midazolam, Diclofenac

Note: ‘/’ refers to none.

Table S2 The change in the use of original and generic drugs in different hospitals in pilot city

Variables	Volume (Ten Thousand DDD)						Expenditure (Ten Thousand CNY)					
	County-level hospitals			City-level hospitals			County-level hospitals			City-level hospitals		
	Pre-	Post-	GR (%)	Pre-	Post-	GR (%)	Pre-	Post-	GR (%)	Pre-	Post-	GR (%)
Policy-list drugs												
Original	58.63	44.80	-23.59	1087.69	1001.03	-7.97	578.28	330.72	-42.81	10440.88	7321.61	-29.88
Generic	319.60	998.58	212.45	1390.06	4870.31	250.37	1928.04	1236.74	-35.86	16835.88	14402.73	-14.45
Bid-winning drugs												
Original	0.00	0.97	/	2.53	27.59	990.51	0.00	1.23	/	11.88	38.29	222.31
Generic	92.63	978.89	956.77	705.82	4511.03	539.12	626.56	1067.55	70.38	8757.28	10636.42	21.46
Non-winning drugs												
Original	58.63	43.83	-25.24	1085.15	973.44	-10.29	578.28	329.49	-43.02	10429.00	7283.32	-30.16
Generic	226.97	19.69	-91.32	684.24	359.28	-47.49	1301.48	169.19	-87.00	8078.60	3766.31	-53.38
Alternative drugs												
Original	473.99	586.91	23.82	2533.25	3297.60	30.17	346.72	489.84	41.28	6676.29	10493.55	57.18
Generic	528.85	515.59	-2.51	1590.56	2108.69	32.58	2373.33	2611.07	10.02	16215.75	18135.71	11.84
Policy-related drugs												
Original	532.62	631.71	18.60	3620.94	4298.63	18.72	925.00	820.56	-11.29	17117.17	17815.16	4.08
Generic	848.45	1514.17	78.46	2980.62	6979.00	134.15	4301.37	3847.81	-10.54	33051.63	32538.44	-1.55

Note: DDD: Defined Daily Dose; CNY, Chinese Yuan; Pre-: March 2018 to November 2018; Post-: March 2019 to November 2019; GR: Growth rate.

Table S3 The impact of the '4+7' policy on the use of original and generic drugs

Variables	Volume (Ten Thousand DDD)				Expenditure (Ten Thousand CNY)			
	Original		Generic		Original		Generic	
	Model 1	Model 2	Model 1	Model 2	Model 1	Model 2	Model 1	Model 2
**Policy-list drugs**								
Treat×Time	-6.06	-11.44	227.38***	215.63***	-171.48*	-212.25*	-148.19*	-200.38**
	(7.43)	(8.72)	(38.86)	(38.02)	(69.94)	(79.05)	(50.00)	(56.06)
Month fixed-effect	Yes	Yes	Yes	Yes	Yes	Yes	Yes	Yes
Hospital attribute fixed-effect	Yes	Yes	Yes	Yes	Yes	Yes	Yes	Yes
R^2	0.273	0.328	0.816	0.825	0.339	0.372	0.293	0.342
**Bid-winning drugs**								
Treat×Time	1.39***	1.00*	261.56**	254.98**	0.76**	-0.75	172.54***	139.13**
	(0.24)	(0.32)	(57.47)	(58.77)	(0.17)	(0.71)	(9.92)	(31.39)
Month fixed-effect	Yes	Yes	Yes	Yes	Yes	Yes	Yes	Yes
Hospital attribute fixed-effect	Yes	Yes	Yes	Yes	Yes	Yes	Yes	Yes
R^2	0.825	0.836	0.826	0.829	0.552	0.603	0.471	0.519
**Non-winning drugs**								
Treat×Time	-7.44	-12.86	-34.41	-38.09	-172.75*	-213.24*	-321.53***	-341.38***
	(7.66)	(9.09)	(20.75)	(21.28)	(69.82)	(78.64)	(51.28)	(56.14)
Month fixed-effect	Yes	Yes	Yes	Yes	Yes	Yes	Yes	Yes
Hospital attribute fixed-effect	Yes	Yes	Yes	Yes	Yes	Yes	Yes	Yes
R^2	0.275	0.323	0.426	0.454	0.341	0.374	0.551	0.566
**Alternative drugs**								
Treat×Time	50.62***	37.84***	13.87	5.71	213.86*	145.92	96.48	34.29

	(3.19)	(7.23)	(10.57)	(10.12)	(87.85)	(88.48)	(59.18)	(30.89)
Month fixed-effect	Yes	Yes	Yes	Yes	Yes	Yes	Yes	Yes
Hospital attribute fixed-effect	Yes	Yes	Yes	Yes	Yes	Yes	Yes	Yes
R^2	0.431	0.469	0.394	0.448	0.384	0.416	0.391	0.441
**Policy-related drugs**								
Treat×Time	44.54***	27.24*	241.25***	223.30***	42.11	-62.27	-51.72†	-155.00*
	(7.06)	(12.30)	(46.76)	(45.73)	(157.74)	(166.99)	(27.45)	(68.72)
Month fixed-effect	Yes	Yes	Yes	Yes	Yes	Yes	Yes	Yes
Hospital attribute fixed-effect	Yes	Yes	Yes	Yes	Yes	Yes	Yes	Yes
R^2	0.409	0.452	0.737	0.755	0.225	0.271	0.371	0.426

Note: DDD: Defined Daily Dose; CNY, Chinese Yuan; Model 1: Crude logistic regression; Model 2: Adjusted logistic regression controlling the confounders; Robust standard error presents in parentheses; *** *P*<0.001, ** *P*<0.01, * *P*<0.05, † *P*<0.1.

Table S4 The impact of the '4+7' policy on the use of drugs in different hospitals

Variables	Volume (Ten Thousand DDD)				Expenditure (Ten Thousand CNY)			
	County-level hospitals		City-level hospitals		County-level hospitals		City-level hospitals	
	Model 1	Model 2	Model 1	Model 2	Model 1	Model 2	Model 1	Model 2
**Policy-list drugs**								
Treat×Time	62.37***	56.08***	194.49***	180.45***	-161.56***	-162.05***	-217.50*	-326.77**
	(3.67)	(7.16)	(1.35)	(7.32)	(14.96)	(24.95)	(81.79)	(84.78)
Month fixed-effect	Yes	Yes	Yes	Yes	Yes	Yes	Yes	Yes
Hospital attribute fixed-effect	Yes	Yes	Yes	Yes	Yes	Yes	Yes	Yes
R^2	0.699	0.733	0.813	0.823	0.534	0.556	0.224	0.269
**Bid-winning drugs**								
Treat×Time	96.46***	91.84***	215.74***	210.30***	39.76***	24.95*	159.35***	131.98***
	(1.26)	(2.63)	(19.59)	(21.50)	(4.53)	(9.27)	(19.54)	(20.83)
Month fixed-effect	Yes	Yes	Yes	Yes	Yes	Yes	Yes	Yes
Hospital attribute fixed-effect	Yes	Yes	Yes	Yes	Yes	Yes	Yes	Yes
R^2	0.864	0.869	0.875	0.880	0.478	0.525	0.421	0.466
**Non-winning drugs**								
Treat×Time	-34.12***	-35.22***	-21.37	-32.15	-201.51***	-188.18***	-376.94***	-456.76***
	(2.50)	(4.73)	(19.30)	(20.10)	(11.61)	(15.97)	(63.50)	(75.53)
Month fixed-effect	Yes	Yes	Yes	Yes	Yes	Yes	Yes	Yes
Hospital attribute fixed-effect	Yes	Yes	Yes	Yes	Yes	Yes	Yes	Yes
R^2	0.566	0.610	0.257	0.304	0.548	0.564	0.419	0.443
**Alternative drugs**								
Treat×Time	-9.08	-15.88*	75.11***	54.11***	-15.39†	-60.40*	332.26***	226.63**

	(5.48)	(5.09)	(7.57)	(8.93)	(8.06)	(26.53)	(42.75)	(68.90)
Month fixed-effect	Yes	Yes	Yes	Yes	Yes	Yes	Yes	Yes
Hospital attribute fixed-effect	Yes	Yes	Yes	Yes	Yes	Yes	Yes	Yes
R^2	0.501	0.520	0.420	0.456	0.572	0.597	0.351	0.382
**Policy-related drugs**								
Treat×Time	53.29***	38.79***	269.59***	234.00***	-176.95***	-214.47***	114.76	-102.08
	(7.57)	(7.17)	(8.14)	(15.25)	(20.91)	(31.90)	(123.90)	(151.41)
Month fixed-effect	Yes	Yes	Yes	Yes	Yes	Yes	Yes	Yes
Hospital attribute fixed-effect	Yes	Yes	Yes	Yes	Yes	Yes	Yes	Yes
R^2	0.596	0.621	0.697	0.715	0.598	0.620	0.209	0.258

Note: DDD: Defined Daily Dose; CNY, Chinese Yuan; Model 1: Crude logistic regression; Model 2: Adjusted logistic regression controlling the confounders; Robust standard error presents in parentheses; *** P<0.001, ** P<0.01, * P<0.05, † P<0.1.

Table S5 The impact of the ‘4+7’ policy on the expenditure of original and generic drugs in different hospitals

Variables	County-level hospitals				City-level hospitals			
	Original		Generic		Original		Generic	
	Model 1	Model 2	Model 1	Model 2	Model 1	Model 2	Model 1	Model 2
**Policy-list drugs**								
Treat×Time	-50.63***	-46.57**	-110.93***	-115.19***	-139.65*	-191.01*	-77.97**	-137.79**
	(7.49)	(9.42)	(8.98)	(17.87)	(58.61)	(67.49)	(24.47)	(29.63)
Month fixed-effect	Yes	Yes	Yes	Yes	Yes	Yes	Yes	Yes
Hospital attribute fixed-effect	Yes	Yes	Yes	Yes	Yes	Yes	Yes	Yes
R^2	0.363	0.380	0.543	0.567	0.268	0.303	0.185	0.229
**Bid-winning drugs**								
Treat×Time	/	/	39.76***	24.92*	0.84**	-0.40	158.03***	128.18***
	/	/	(4.52)	(9.14)	(0.13)	(0.38)	(19.38)	(20.95)
Month fixed-effect	Yes	Yes	Yes	Yes	Yes	Yes	Yes	Yes
Hospital attribute fixed-effect	Yes	Yes	Yes	Yes	Yes	Yes	Yes	Yes
R^2	/	/	0.477	0.524	0.608	0.640	0.419	0.462
**Non-winning drugs**								
Treat×Time	-50.63***	-46.53**	-150.88***	-140.49***	-140.96*	-191.98*	-236.09***	-266.26***
	(7.48)	(9.31)	(5.38)	(9.82)	(58.43)	(67.32)	(6.32)	(13.12)
Month fixed-effect	Yes	Yes	Yes	Yes	Yes	Yes	Yes	Yes
Hospital attribute fixed-effect	Yes	Yes	Yes	Yes	Yes	Yes	Yes	Yes
R^2	0.362	0.379	0.613	0.626	0.270	0.305	0.484	0.498
**Alternative drugs**								

Treat×Time	-4.25	-29.87†	-11.13	-30.45	220.89*	164.98†	111.28†	61.74†
	(5.63)	(16.15)	(6.99)	(17.50)	(91.93)	(80.29)	(52.00)	(32.13)
Month fixed-effect	Yes	Yes	Yes	Yes	Yes	Yes	Yes	Yes
Hospital attribute fixed-effect	Yes	Yes	Yes	Yes	Yes	Yes	Yes	Yes
R^2	0.373	0.427	0.565	0.588	0.364	0.386	0.252	0.280
**Policy-related drugs**								
Treat×Time	-54.89**	-76.44**	-122.07***	-145.64***	81.35	-24.63	33.31	-76.05
	(13.02)	(19.09)	(11.77)	(19.70)	(150.41)	(146.40)	(33.01)	(50.07)
Month fixed-effect	Yes	Yes	Yes	Yes	Yes	Yes	Yes	Yes
Hospital attribute fixed-effect	Yes	Yes	Yes	Yes	Yes	Yes	Yes	Yes
R^2	0.433	0.458	0.597	0.616	0.147	0.187	0.226	0.270

Note: Model 1: Crude logistic regression; Model 2: Adjusted logistic regression controlling the confounders; Robust standard error presents in parentheses; *** P<0.001, ** P<0.01, * P<0.05, † P<0.1.

Table S6 The in-time placebo test on the impact of the '4+7' policy on the use of drugs

Variables	Volume (ten thousand DDD)		Expenditure (ten thousand CNY)	
	Model 1	Model 2	Model 1	Model 2
**Policy-list drugs**				
Treat×Time	116.62***	46.96	48.18	156.00*
	(17.13)	(27.78)	(58.38)	(55.28)
Month fixed-effect	Yes	Yes	Yes	Yes
Hospital attribute fixed-effect	Yes	Yes	Yes	Yes
R^2	0.426	0.596	0.233	0.315
**Bid-winning drugs**				
Treat×Time	121.70***	39.48	159.22***	108.94***
	(24.92)	(29.16)	(9.65)	(20.87)
Month fixed-effect	Yes	Yes	Yes	Yes
Hospital attribute fixed-effect	Yes	Yes	Yes	Yes
R^2	0.328	0.526	0.460	0.518
**Non-winning drugs**				
Treat×Time	-4.89	6.95	-112.11†	43.10
	(8.65)	(4.94)	(54.82)	(59.40)
Month fixed-effect	Yes	Yes	Yes	Yes
Hospital attribute fixed-effect	Yes	Yes	Yes	Yes
R^2	0.221	0.304	0.183	0.309
**Alternative drugs**				
Treat×Time	46.51**	20.12	242.87***	120.19*
	(13.36)	(15.22)	(18.37)	(41.20)
Month fixed-effect	Yes	Yes	Yes	Yes
Hospital attribute fixed-effect	Yes	Yes	Yes	Yes
R^2	0.437	0.496	0.402	0.467
**Policy-related drugs**				
Treat×Time	163.13***	67.35	291.05**	280.79**
	(30.16)	(41.32)	(51.59)	(63.45)
Month fixed-effect	Yes	Yes	Yes	Yes
Hospital attribute fixed-effect	Yes	Yes	Yes	Yes
R^2	0.466	0.584	0.370	0.404

Note: DDD: Defined Daily Dose; CNY, Chinese Yuan; Model 1: Crude logistic regression; Model 2: Adjusted logistic regression controlling the confounders; Robust standard error presents in parentheses; *** *P*<0.001, ** *P*<0.01, * *P*<0.05.

Table S7 The in-space placebo test on the impact of the ‘4+7’ policy on the use of drugs

Variables	Volume (ten thousand DDD)		Expenditure (ten thousand CNY)	
	Model 1	Model 2	Model 1	Model 2
**Policy-list drugs**				
Treat×Time	-5.16	-4.29	-40.90	-29.54
	(5.13)	(3.30)	(24.93)	(17.37)
Month fixed-effect	Yes	Yes	Yes	Yes
Hospital attribute fixed-effect	Yes	Yes	Yes	Yes
R^2	0.418	0.497	0.362	0.425
**Bid-winning drugs**				
Treat×Time	-3.77†	-2.05	-19.96†	-11.92
	(2.01)	(1.63)	(10.40)	(10.24)
Month fixed-effect	Yes	Yes	Yes	Yes
Hospital attribute fixed-effect	Yes	Yes	Yes	Yes
R^2	0.315	0.424	0.303	0.390
**Non-winning drugs**				
Treat×Time	-1.53	-2.53	-21.83	-13.88
	(3.42)	(3.00)	(17.71)	(21.54)
Month fixed-effect	Yes	Yes	Yes	Yes
Hospital attribute fixed-effect	Yes	Yes	Yes	Yes
R^2	0.407	0.469	0.335	0.385
**Alternative drugs**				
Treat×Time	-12.16	-10.28	-36.03	-11.14
	(6.71)	(7.88)	(20.27)	(12.71)
Month fixed-effect	Yes	Yes	Yes	Yes
Hospital attribute fixed-effect	Yes	Yes	Yes	Yes
R^2	0.412	0.467	0.478	0.557
**Policy-related drugs**				
Treat×Time	-17.32	-14.16	-76.93	-71.77
	(10.93)	(8.49)	(44.47)	(31.56)
Month fixed-effect	Yes	Yes	Yes	Yes
Hospital attribute fixed-effect	Yes	Yes	Yes	Yes
R^2	0.448	0.510	0.455	0.525

Note: DDD: Defined Daily Dose; CNY, Chinese Yuan; Model 1: Crude logistic regression; Model 2: Adjusted logistic regression controlling the confounders; Robust standard error presents in parentheses; † P<0.1.

Table S8 The mixed placebo test on the impact of the '4+7' policy on the use of drugs

Variables	Volume (ten thousand DDD)		Expenditure (ten thousand CNY)	
	Model 1	Model 2	Model 1	Model 2
**Policy-list drugs**				
Treat×Time	-5.16	-4.28	-40.90	-29.54
	(5.13)	(3.03)	(24.93)	(17.37)
Month fixed-effect	Yes	Yes	Yes	Yes
Hospital attribute fixed-effect	Yes	Yes	Yes	Yes
R^2	0.418	0.497	0.362	0.425
**Bid-winning drugs**				
Treat×Time	-3.77†	-2.05	-19.96†	-11.92
	(2.01)	(1.63)	(10.40)	(10.24)
Month fixed-effect	Yes	Yes	Yes	Yes
Hospital attribute fixed-effect	Yes	Yes	Yes	Yes
R^2	0.315	0.424	0.303	0.390
**Non-winning drugs**				
Treat×Time	-1.53	-5.23	-21.83	-13.88
	(3.42)	(3.00)	(17.71)	(21.54)
Month fixed-effect	Yes	Yes	Yes	Yes
Hospital attribute fixed-effect	Yes	Yes	Yes	Yes
R^2	0.407	0.469	0.335	0.385
**Alternative drugs**				
Treat×Time	-12.16	-10.28	-36.03	-11.14
	(6.71)	(7.88)	(20.27)	(12.71)
Month fixed-effect	Yes	Yes	Yes	Yes
Hospital attribute fixed-effect	Yes	Yes	Yes	Yes
R^2	0.411	0.467	0.478	0.557
**Policy-related drugs**				
Treat×Time	-17.32	-14.16	-76.93	-51.77
	(10.93)	(8.29)	(44.47)	(31.56)
Month fixed-effect	Yes	Yes	Yes	Yes
Hospital attribute fixed-effect	Yes	Yes	Yes	Yes
R^2	0.448	0.510	0.455	0.525

Note: DDD: Defined Daily Dose; CNY, Chinese Yuan; Model 1: Crude logistic regression; Model 2: Adjusted logistic regression controlling the confounders; Robust standard error presents in parentheses; † *P*<0.1.

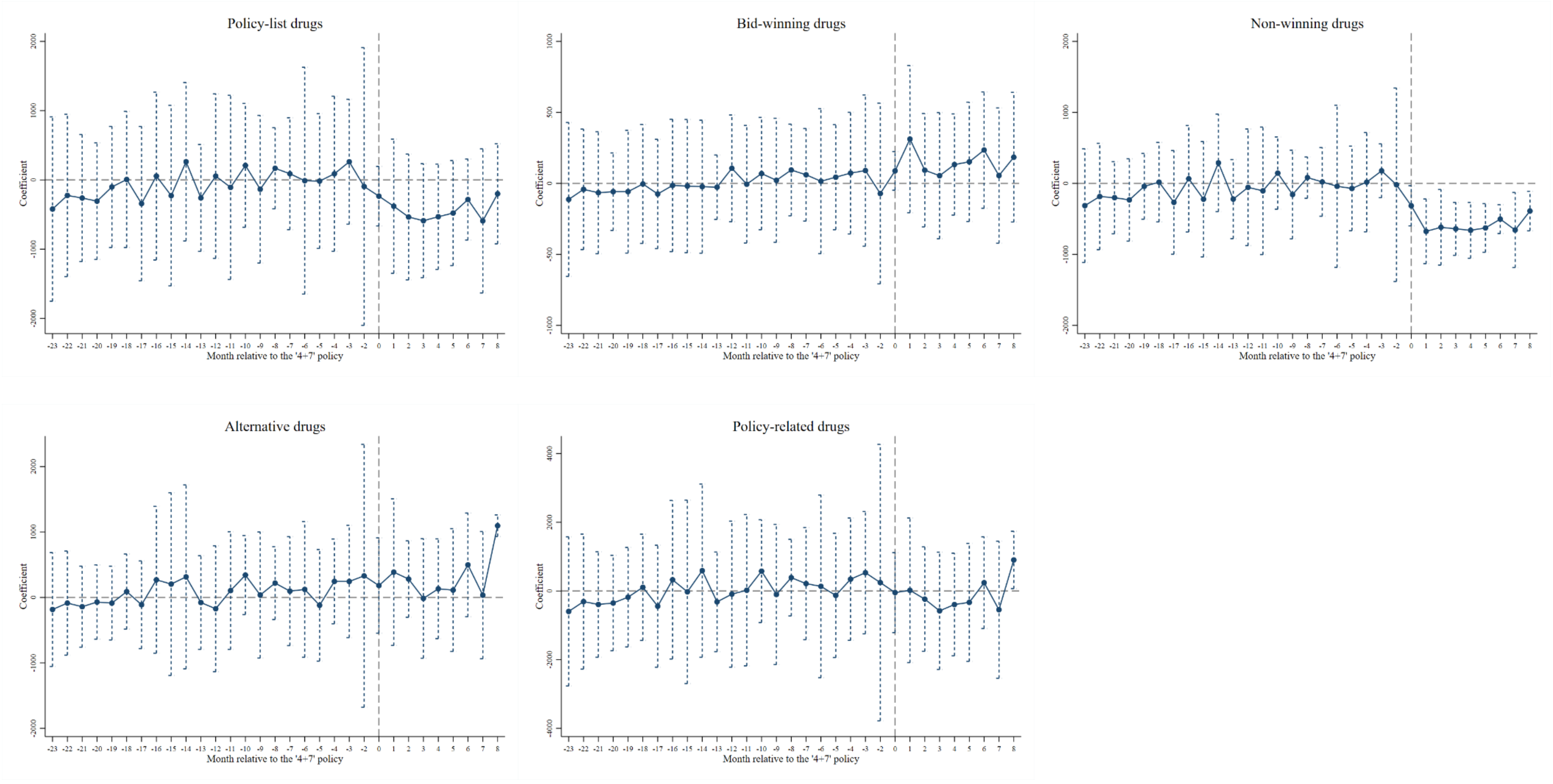

Figure S1 Parallel trends test on the impact of the ‘4+7’ policy on the expenditure of drugs
